# A proposed nomenclature for spinal imaging and interventional procedural reporting

**DOI:** 10.1016/j.inpm.2022.100082

**Published:** 2022-03-17

**Authors:** Jatinder S. Gill, Steven P. Cohen, Thomas T. Simopoulos, Michael B. Furman, Salim M. Hayek, Koen Van Boxem, David J. Kennedy, W. Michael Hooten, Vinil Shah, Milan P. Stojanovic

**Affiliations:** a BIDMC, Harvard Medical School, 330 Brookline Avenue, Boston, MA, 02215, USA; b Johns Hopkins School of Medicine, USA; c BIDMC, Harvard Medical School, USA; d Interventional Spine and Sports Fellowship, OSS Health, Temple University, USA; e Case Western Reserve University, Cleveland, OH, USA; f Department of Anesthesiology, Critical Care and Multidisciplinary Pain Center, Ziekenhuis Oost-Limburg, Lanaken, Genk, Belgium; g Department of Anesthesiology and Pain Medicine, Maastricht University Medical Center, Maastricht, the Netherlands; h Department of Physical Medicine and Rehabilitation, Vanderbilt Center for Musculoskeletal Research, USA; i College of Medicine, Mayo Clinic, Rochester, MN, USA; j Department of Radiology and Biomedical Imaging, University of California San Francisco, USA; k VA Boston Healthcare, Edith Nourse Rogers Memorial VA Hospital, Harvard Medical School, Boston, MA, USA

## Abstract

**Objective:**

To develop precise universal standard interventional spine nomenclature for reporting procedural details and anatomy.

**Methods:**

There is no comprehensive nomenclature of spinal imaging anatomy that can be used for anatomical and procedural reporting. Given this critical lack of unifying terminology, a system of nomenclature was developed de novo by expert consensus, based upon clinical needs, and previously published reports.

**Results:**

Nomenclature for anatomical and spine procedural reporting for interlaminar and transforaminal approaches was developed using zones in each view. Separate nomenclature for medial branch procedural reporting and discs and vertebral body location and procedural reporting is also presented.

**Conclusion:**

There is a need for a unified anatomical location reporting system in interventional spine. The first step is the development of a precise, simple, and intuitive nomenclature, as reported here. The second is ratification followed by dissemination and adoption in clinical practice.

## 1 Introduction

There has been explosive growth and development in the field of interventional spine medicine. Practitioners of contemporary interventional spine span many specialties and belong to multiple educational and professional societies. Common spine interventions include various types of epidural steroid injections (ESI), medial branch blocks (MBB) and radiofrequency ablation (RFA), spinal cord stimulation, intrathecal drug delivery, minimally invasive lumbar decompression, interspinous spacers, vertebral augmentation, percutaneous discectomy, basivertebral nerve ablation, minimally invasive spine surgery, and regenerative medicine, amongst others. Given the diversity of specialties, societies, and procedures, myriad formal and informal names and colloquiums exist for describing spinal anatomy. Some terms are historic, some are geographically-based, some are specialty- or society-specific, and others may even be institutionally-based.

In this manuscript we propose a new, standardized method for reporting spinal anatomy – an interventional spine “dictionary”. Homogenized nomenclature should be intuitive and easy to understand, with implementation critical for four important reasons:

First, outcomes of spine interventions are correlated with technical parameters [[1], [2], [3]]. For ESI, needle location determines contrast and injectate flow patterns, while electrode position during RFA may affect the probability of capturing the target nerve [[1], [2], [3], [4], [5], [6], [7], [8]]. However, reporting is often ambiguous because of difficulties in communicating the exact anatomical location (e.g., parasagittal interlaminar ESI may result in different spread patterns and outcomes depending on whether the needle tip is placed in a far lateral position vs. just off midline). This is more likely to occur with poor methodological descriptions since the term ‘parasagittal’ does not convey distance from midline. Thus, when reporting findings of research studies that involve anatomic precision, it is imperative that the system can unambiguously communicate precise location to facilitate reproducibility in future studies or clinical practice.

Second, location of needle tip is a critical determinant of safety. In an analysis of ASA closed claims database for cervical pain treatments it was revealed that a majority of injuries were related to direct needle trauma to nerves or the spinal cord [9]. By creating a homogenized nomenclature such as reported here, safety can be enhanced by clearly describing red zones where neural trauma may occur.

Third, a precise method of communicating location will assist the interventionalist in replicating a procedure. For example, if an epidural needle is placed in Zone 3 in an antero-posterior (AP) view, it delivers a very clear picture compared to using vague terminology such as parasagittal, paramedian, gutter, paraforaminal, off-midline, and lateral recess, none of which provide information on the distance from the midline.

Fourth, there is a need to reduce the amount of time spent writing and documenting a procedure, with studies demonstrating that physicians typically spend over one-third of their workday performing administrative tasks [10]. A universally adopted system has the potential to reduce the workload of documentation while improving descriptive precision. The language clutter that currently exists is readily apparent by examining the terms used for transforaminal ESI such as ‘safe triangle’ ‘retroneural’, ‘supraneural’, ‘infraneural’, ‘pre-ganglionic’, ‘post-ganglionic’, ‘Kambin triangle’, ‘modified safe triangle’, and ‘retrodiscal’.

The need for standardized terminology for interventional spine care is manifest, and uniform nomenclature is used in pain medicine's umbrella specialties, including the American Society of Anesthesiology (ASA) physical status and airway classification in anesthesia, Glasgow coma scale in neurology, and terminology for classifying disc and endplate pathology in interventional spine and radiology [[11], [12], [13], [14], [15]]. One of the reasons for the lack of any unifying lexicon in spine is the diversity of practitioners and specialties. Previously, we proposed standardizing nomenclature for spine interventionalists, but until now this has yet to be systematically undertaken [16].

## 2 Methods

In designing the lexicon, consideration was given to classification based upon the views of experts, published literature, and needs-based assessments from anatomical and procedural perspectives. Common interventions involving epidural access, or requiring proximity to the epidural space, include ESI (interlaminar or transforaminal), percutaneous spinal cord stimulator (SCS) placement, intrathecal drug delivery systems (IDDS), and minimally invasive spine interventions such as interspinous spacers or minimally invasive lumbar decompression (MILD). For all of these, describing the needle tip location in an AP view and the depth of insertion using contralateral oblique or lateral views is important. We created a system similar to traffic lights going from green to red to denote areas of safety and areas of caution.

For medial branch blocks (MBB), and RFA, reporting needs are unique to the procedure and a separate system is described. Similarly, for disc and vertebral body interventions, we developed a distinct nomenclature suitable to precisely describe needle tip position and relevant components of disc (annulus fibrosus vs. nucleus pulposus) and vertebral body anatomy.

## 3 Results

**Fig. 1:**
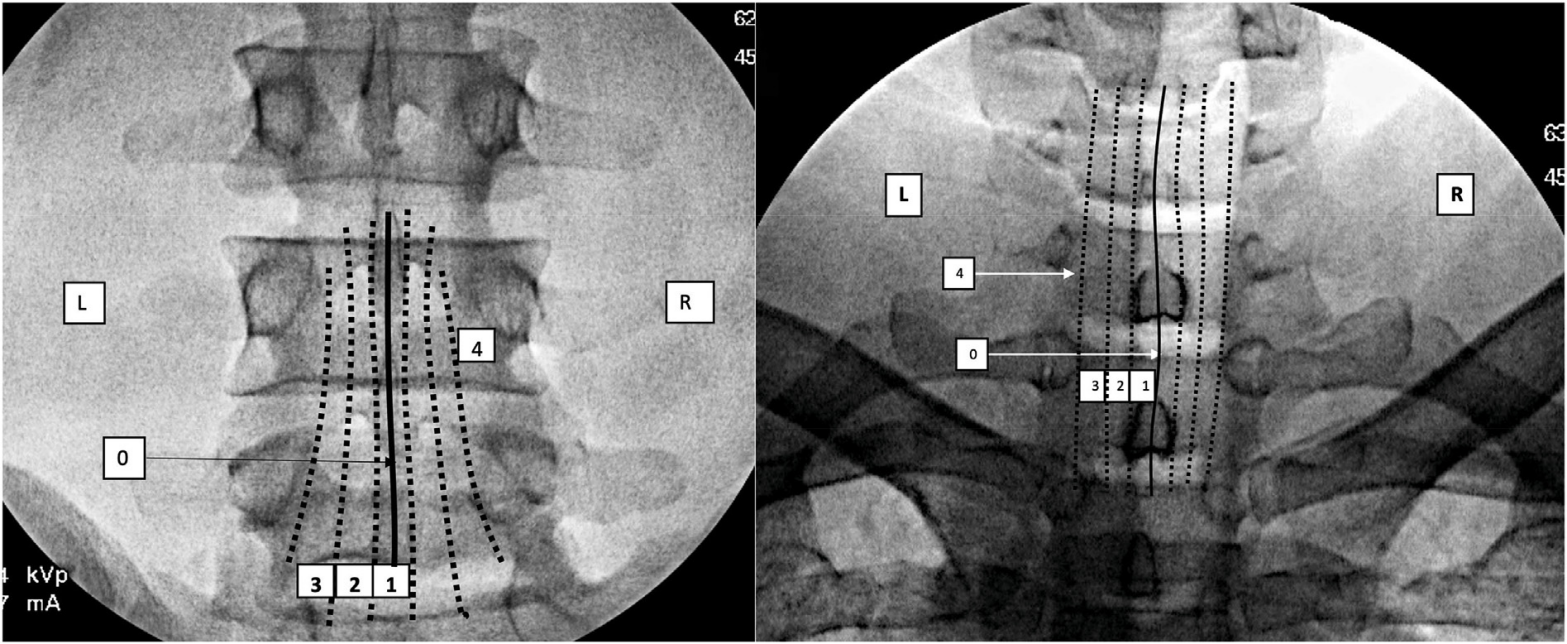
Zones in AP view L – left, R-right.

**Fig. 2:**
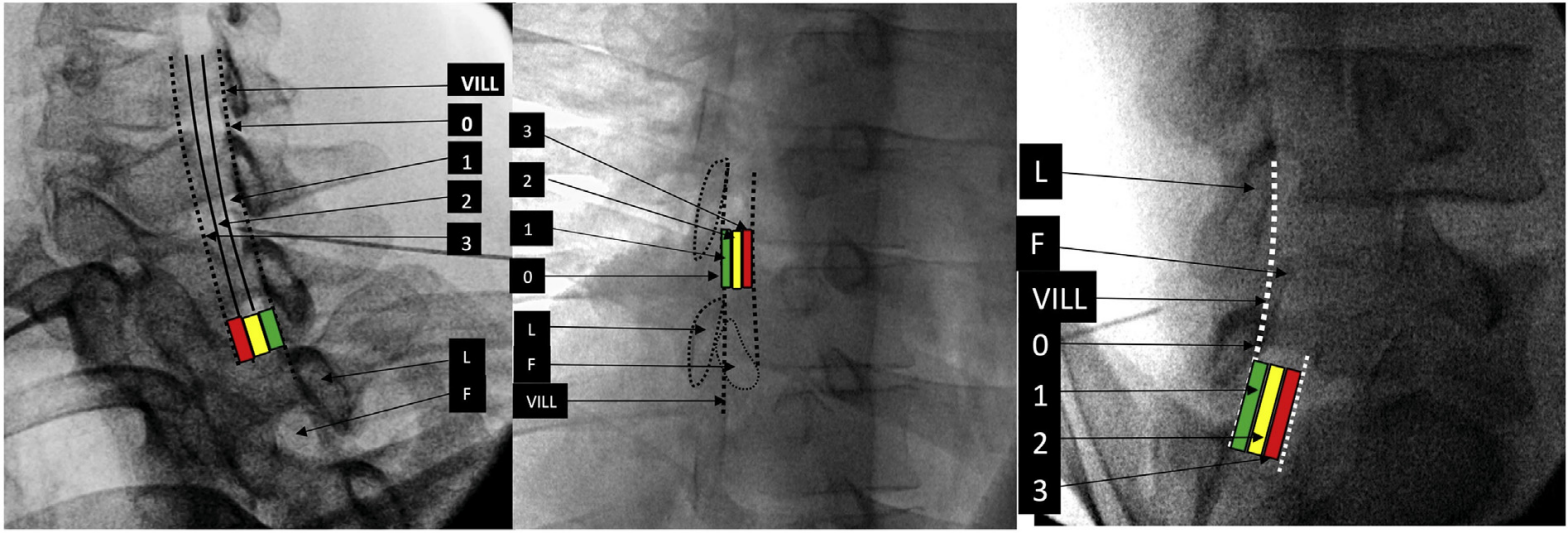
Zones in CLO view Cervical, Thoracic and Lumbar. VILL – ventral interlaminar line, L – lamina, F - foramen.

**Fig. 3:**
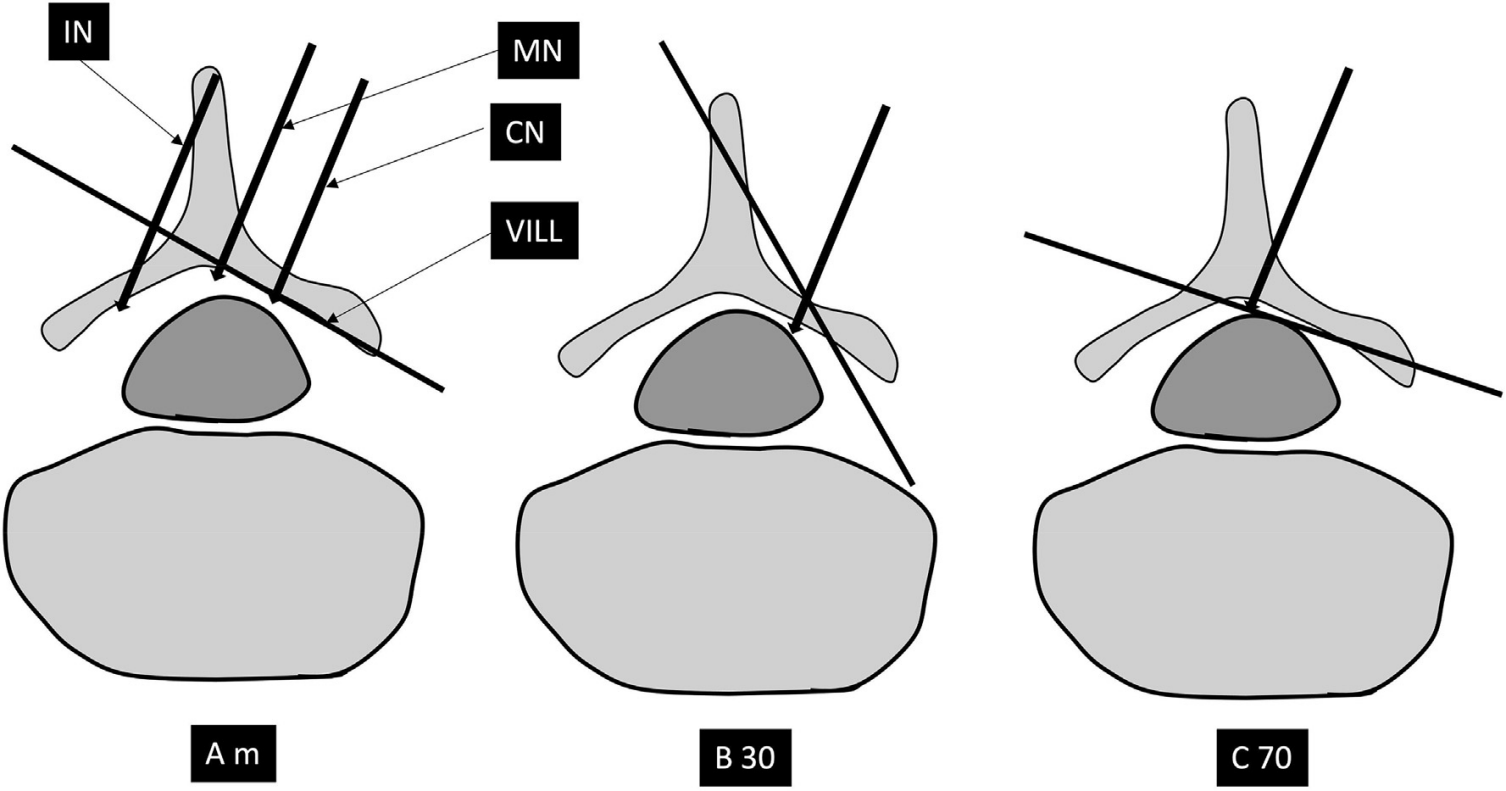
When the fluoroscope is parallel to the angle of the lamina at the measured angle of obliquity (Am), the needle tip is visualized as soon as it breaches the ventral interlaminar margin, unless there is ligamentum flavum hypertrophy then the needle tip will appear more anterior. Note the relationship of the needle tip with the VILL. Note that midline (MN) needle tip may appear slightly more anterior, but the depth of needle tip is independent of medio-lateral orientation at the measured angle of obliquity. The mediolateral location can only be determined in the AP view. Obtaining correct sagittal trajectory prior to switching to CLO view is important. The needle tip location may be checked intermittently in AP view, and prior to entering the epidural space so as to assure that the correct region is accessed. The ipsilateral needle (IN) will appear very anterior. With lesser obliquity (from AP) at 30° (B30), the lamina is transected by the line of view and all midline and paramedial needle tips appear significantly anterior to the VILL. Note that with over-obliquity to 70°, the laminar cross section is not well visualized and a needle tip in the epidural space may appear posterior to the VILL. Also, independence of needle tip location from medio-lateral insertion point is lost when the line of view is not parallel to the lamina. IN - ipsilateral needle, MN – midline needle, CN – contralateral needle, VILL – ventral interlaminar line. A m – contralateral oblique (CLO) at measured angle, B 30 – CLO at 30°, C70 – CLO at 70°.

**Fig. 4:**
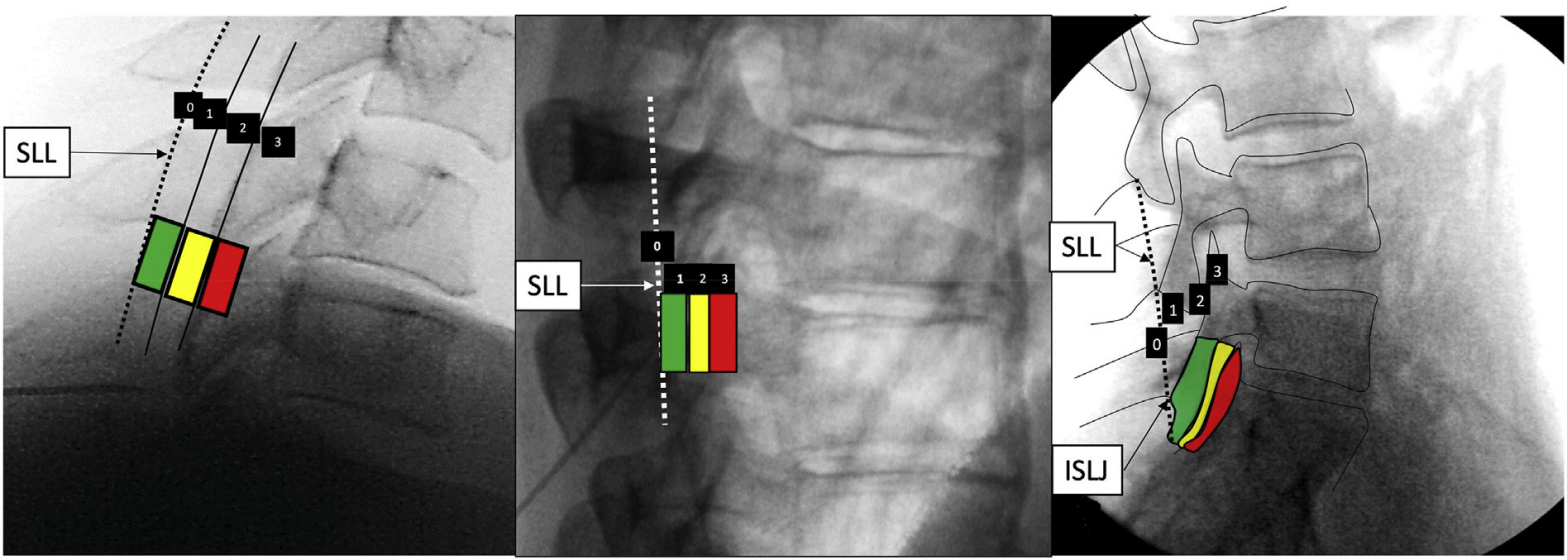
Zones in Lateral view, Cervical, Thoracic and Lumbar. SLL – spinolaminar junction, not well visualized but lies slightly behind the facet lucency. ISLJ - inferior spinolaminar junction is the earliest a needle tip may enter the epidural space.

For procedures involving interlaminar epidural access or proximity to the dorsal epidural space as discussed above, we created zones in each of the views based upon anatomical details that were described in previous publications but have been modified for uniformity [[17], [18], [19]]. We developed a system where for interlaminar access each view has 4 zones numbered from 0 to 3 (Fig. 1, Fig. 2, Fig. 3, Fig. 4). In the AP view, Zone 0 represents the midline and Zone 3 the most lateral part of the interlaminar opening. In the contralateral oblique (CLO) and lateral views, Zone 0 represents the point proximal to which there is no possibility of the needle entering the epidural space while Zone 3 represents the maximum depth at which dorsal epidural space may be accessed. The rationale for four zones is that it provides descriptive precision without unnecessary complexity. The AP zone is reported first, followed by CLO, followed by lateral. Although it is preferable that all visualized needle tip positions are reported, in case of missing or unobtained views, either one, two, or all three may be reported using the correct sequence.

**Fig. 5:**
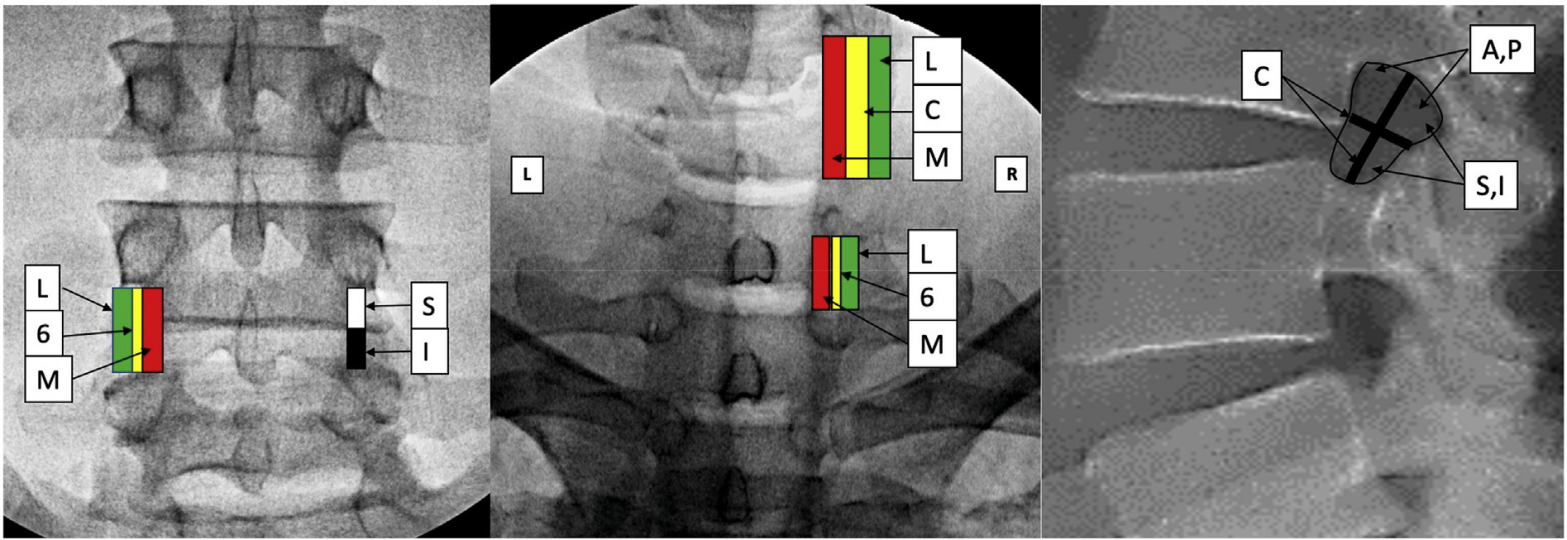
Zones for transforaminal procedural reporting AP Lumbar, AP cervical and thoracic and Lateral Lumbar. Zone “C” may be reported in the cephalo-caudad and antero-posterior dimensions when there is ambiguity created by a location that cannot be assigned. L – lateral, M− medial, C – central, S – superior, I – inferior, A – anterior, P – posterior.

An oblique (i.e., transforaminal) approach is used not only for transforaminal epidural steroid injections (TFESI), but also for minimally invasive disc access and minimally invasive surgical procedures such as percutaneous discectomy. In the AP view, the needle tip position under the pedicle was divided into the middle fifth representing the 6:00 O'clock position and designated as “6”, with areas lateral to it designated as “L” and medial to it designated as “M”. In the lateral view, in the cephalo-caudad plane, the upper half is designated as superior or “S” while the lower half is labeled as inferior or “I”. In the antero-posterior dimension in a true lateral view, the anterior half is designated “A” and posterior half is “P” (Fig. 5). Thus, the needle tip position may be described first in the AP view, then in cephalo-caudad dimension in lateral view, followed by the anteroposterior dimension in lateral view; either one, two, or all three locations may be reported in the correct sequence, although it is preferable that all visualized views and dimensions are reported. Aside from the AP view, we did not create a middle zone to minimize complexity. When the needle position is intermediate in lateral view, best judgement should be employed to select one quadrant over the other or the term central, “C” may be used (Fig. 5).

**Fig. 6:**
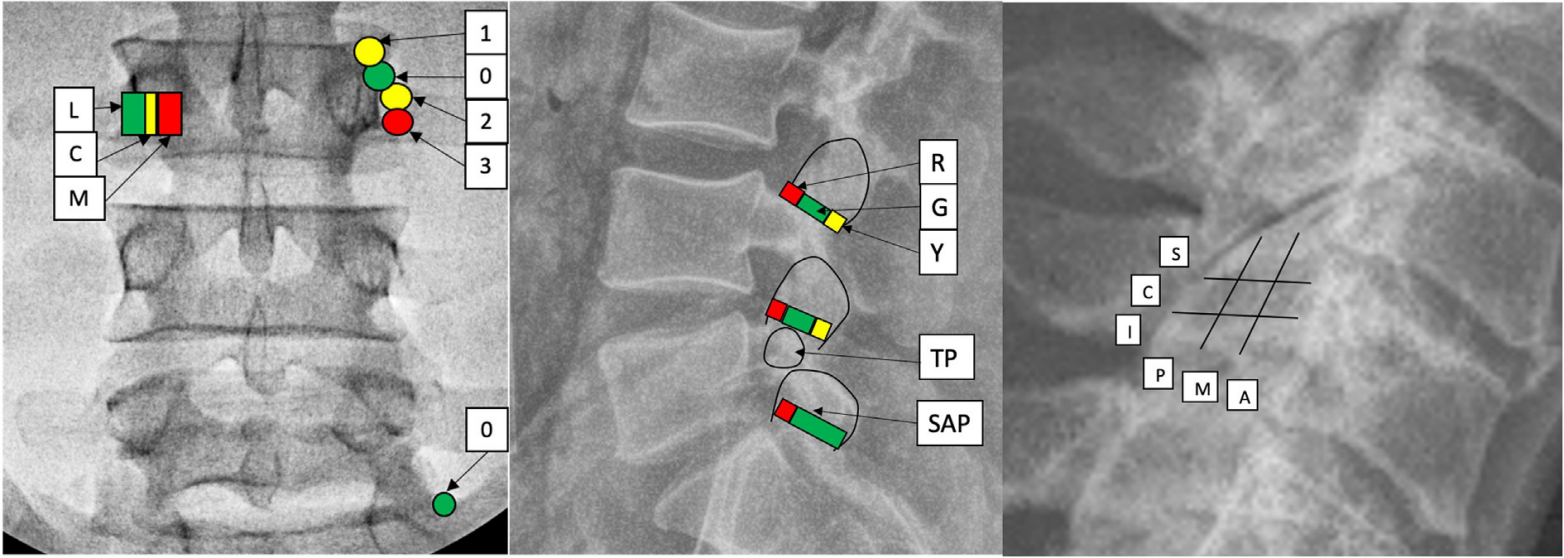
System for reporting MBB and RFA. L – lateral, C – central, M −medial, R – red, G – green, Y – yellow, S – superior, I – inferior, P – posterior, M -middle, A – anterior, TP – transverse process, SAP – superior articular process. (For interpretation of the references to color in this figure legend, the reader is referred to the Web version of this article.)

For the reporting of lumbar medial branch blocks and radiofrequency procedures, the most important landmarks are the superior articular process and the transverse process. In the AP view, four regions were created to describe the cephalo-caudad needle tip location from 0 to 3 and three zones were created to describe the needle tip location in mediolateral orientation. In the lateral view three zones representing anterior quarter, middle half, and posterior quarter (except for L5 dorsal ramus) were created in relation to the superior articular process. The cephalo-caudad location is reported first, followed by the mediolateral designation, followed by lateral (Fig. 6). Either one, two, or all three may be reported using the correct sequence, although it is preferable that needle tip position in both AP and lateral views be reported.

For the cervical MB and RFA reporting, the lateral view with articular pillars superimposed is most reproducible, and the needle tip location may be described first in the cephalo-caudad dimension and second in the AP dimension, dividing the articular pillar into three zones in each dimension (Fig. 6). For thoracic reporting the variability of medial branches dictates that the procedure be reported individually.

**Fig. 7:**
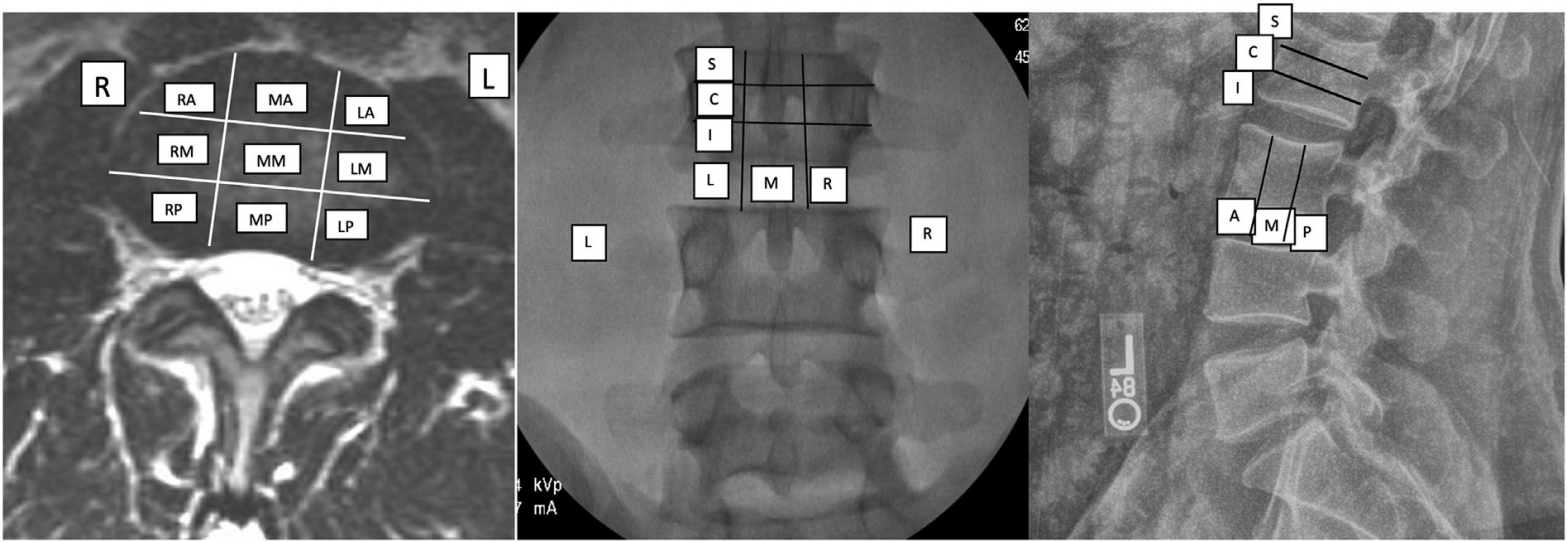
Zones for disc and vertebral body reporting, lumbar MRI, lumbar AP radiograph (right and left are flipped) and lumbar lateral radiograph. R – right, M −middle (both AP and lateral dimension), L-left, A-anterior, P – posterior, S- superior, C- central, I - inferior.

The disc can be divided into thirds both in AP and lateral views, giving rise to 9 segments (Fig. 7). The vertebral body can be divided into a further 3 cephalo-caudad segments: superior, central and inferior. The AP zone is reported first, the anteroposterior dimension in true lateral view is reported second, and the cephalo-caudad is reported third and only used for vertebral body descriptions.

## 4 Discussion

In the following paragraphs, we describe the nomenclature in further detail.

### 4.1 AP view zones for interlaminar procedural and anatomical reporting (Fig. 1, Table 1)

AP view is the gold standard for defining spine location in medio-lateral and cephalo-caudad directions. The interlaminar area presents a wide target that can be accessed at multiple points (Fig. 1). Therefore, it is imperative that the precise location be described so the same position can be replicated by clinician colleagues and researchers. In a true AP view, the spinous processes lie midway between the pedicles in the lumbar and thoracic regions, and midway between the articular pillars in the cervical spine. The mediolateral location of the needle is therefore definable. If the endplates are aligned, then the cephalo-caudal location is also clearly definable. AP view may be reported as such, or further qualified if the endplates are aligned. Interventions that can use this nomenclature for reporting AP views include:

- Interlaminar Epidural Injection – needle and contrast reporting
- Spinal Cord Stimulator Lead Placement
- Interspinous Spacers
- Minimally Invasive Lumbar Decompression
- Radiographic Reporting
- Other interventions requiring epidural/intrathecal access such as biologics and intrathecal needle placement
- Interlaminar Minimally Invasive Spine Surgery

**Table 1:** Zones in AP, CLO, and lateral view (interlaminar epidural access and other relevant procedures). VILL – ventral interlaminar line. For interlaminar epidural: AP view location is reported first, CLO view second, lateral view third. Whichever is not reported may be marked by an X in the three letters.

	Zone 0 (midline)	Zone 1	Zone 2	Zone3
AP View Zones	midline	Within the boundaries of spinous process, right and left	Medial half of region between spinous process margin and lateral edge of interlaminar opening, right and left	Lateral half of region between spinous process and lateral edge of interlaminar opening, right and left
CLO View Zones (cervical)	On the Ventral Interlaminar Line	Posterior third of the space bounded by the VILL and the uncinate line (Green)	Middle third of the space bounded by the VILL and the uncinate line (Yellow)	Anterior third of the space bounded by the VILL and the uncinate line (Red)
CLO View Zones (Lumbar and thoracic)	On the Ventral Interlaminar Line	Posterior third of the space bounded by the VILL posteriorly and the boundary of the visualized foramen anteriorly (Green)	Middle third of the space bounded by the VILL posteriorly and the boundary of the visualized foramen anteriorly (Yellow)	Anterior third of the space bounded by the VILL posteriorly and the boundary of the visualized foramen anteriorly (Red)
Lateral View Zones (cervical)	On the spinolaminar junction	Posterior half of the area between the spinolaminar line and posterior articular pillar line. (Green)	Anterior half of the area between the spinolaminar line and posterior articular pillar line. (Yellow)	Anterior to the posterior articular pillar line (Red)
Lateral View Zones (Lumbar & Thoracic)	On the spinolaminar junction	From spinolaminar line to the facet lucency (Green)	In the facet lucency (Yellow)	Anterior to the facet lucency (Red)

In an AP view, the true midline in the center of the spinous process spanning the horizontal plane is Zone 0.

The area on either side of Zone 0, but within the confines of the spinous process lateral margins is Zone 1. The area between the lateral margins of the spinous process and the most lateral parts of the interlaminar opening is divided into two equal zones, Zone 2 medially and Zone 3 laterally. Any area lateral to the interlaminar opening but medial to the medial margin of the pedicle is Zone 4, which may or may not be present.

### 4.2 CLO view zones for interlaminar procedures and other relevant procedures using this anatomy (Fig. 2, Fig. 3, Table 1)

The CLO view can facilitate or enable needle tip visualization, help plan needle trajectory, as well as underscore the clear bony landmarks that serve as location markers for the posterior boundary of the epidural space [[17], [18], [19], [20], [21]]. During CLO views, the image intensifier is angled contralateral to the needle tip, about 50° in the cervical spine, about 45° in lumbar spine, and about 60° in the mid-thoracic spine, thereby accentuating the identification of the lamina and an imaginary line that joins the ventral margin of the laminae, a.k.a. the Ventral Interlaminar Line (VILL, Fig. 2, Fig. 3) [[17], [18], [19]]. The angle is measured with the true AP position deemed to be zero degrees. For midline approaches, the fluoroscope may be obliqued to either direction. Entry of the needle tip into the epidural space occurs at the VILL or slightly deeper. The degree of angulation determines the needle tip location. If the angulation is less than recommended, the needle tip position will appear deeper, whereas excessive angulation may lead to the needle entering the epidural space before the VILL [17,18] (Fig. 3). The most important concepts in using the contralateral view are to make sure that the needle tip is contralateral to the angulation, to use the recommended or measured angulation, and to use contrast to determine if the needle tip has already reached the epidural space after the VILL is breached and there is no loss of resistance. This is especially important in the thoracic and cervical spine, where ligamentum flavum hypertrophy is less likely, and the risks of excessive advancement may be grave. In the cervical spine at the appropriate angulation, most needle tips will appear at the VILL or slightly in front of it when the epidural space is entered [17] (Fig. 3). Some midline needle tips may appear slightly deeper (<2.5 mm in front of VILL) in the cervical spine. This may relate to the fact that the angle of the lamina is flatter towards the midline, there is some depth for the needle to move in the epidural space, and the needle may have veered ipsilaterally [17] (Fig. 3). If the needle tip traverses ipsilateral to the angulation, then it will appear dangerously deep (Fig. 3). In the thoracic spine most needle tips will appear at the VILL or slightly anterior [19]. In the lumbar spine the needle tip may be deeper to the VILL, and this usually occurs with ligamentum flavum hypertrophy [18]. Entry at the VILL represents Zone 0 in all three regions of the spine. In the cervical spine, zones are formed by dividing the area between the VILL and the line joining the uncinate processes into three equal zones in a posterior to anterior direction, correspondingly named Zones 1 to 3 [17]. In the lumbar and thoracic regions, the same sequence is followed; however, the anatomy is slightly different, namely the anterior border is formed by the anterior margin of the foramina. The area between the VILL posteriorly and the anterior margin of the observed foramen may be divided into three equal zones posteriorly to anteriorly. The posterior boundary of Zone 1 is always represented by the VILL in all areas of the spine irrespective of whether the posterior boundary of the foramen overlaps with the VILL [17]. The zones are color-coded like traffic lights during the transition from posterior to anterior, with green (go) representing Zone 1, yellow (caution) representing Zone 2, and red (stop and reassess) representing Zone 3. In the cervical spine, entry into the epidural space most often occurs in Zone 0 or Zone 1, represented by the green area in Fig. 2. Occasionally the needle may reach Zone 2; this is sometimes observed with midline needles [17] (Fig. 3). In the thoracic spine, at 60° angulation from the AP plane, the loss occurs most often at the VILL or slightly in front of it [19]. In the lumbar spine at 45°, the loss occurs at the VILL or in front of it when there is ligamentum flavum hypertrophy. Larger degrees of angulation is not recommended as the epidural space can be entered before the VILL, though it will still be behind the dura mater and does not create an unsafe situation [18].

### 4.3 Lateral view zones (epidural access and other procedures relying on the location of the epidural space, Fig. 4, Table 1)

The lateral view is the reference standard for defining the posterior boundary of the vertebral body, the boundaries of the spinal canal, as well as the antero-posterior location during disc and vertebral body interventions. The lateral view can provide an estimate of the true posterior boundary of the epidural space; however, it is not as precise as that afforded by the CLO view since the boundary of the posterior epidural space is curvilinear and there is a topographic mismatch [17]. This is one reason for the wide range that exists for the location of the epidural space as seen in the lateral view [17,18]. Ligamentum flavum hypertrophy, commonly found in the lumbar spine in elderly patients, can also lead to deeper loss-of-resistance as visualized both in CLO and lateral views [18]. The zones are again color-coded like traffic lights as one transitions posteriorly to anteriorly with green (go) representing Zone 1, yellow (caution) representing Zone 2, and red (stop and reassess) representing Zone 3.

In the cervical spine, the most proximate point at which epidural space may be entered is at the spinolaminar junction, in Zone 0 (Fig. 4). More commonly, loss of resistance is appreciated between the spinolaminar line and the posterior articular pillar line. This area is divided into two equal parts from posterior to anterior in an antero-posterior plane- Zone 1 and Zone 2, respectively. Anything anterior to Zone 2 represents Zone 3; this is less likely to be encountered and may signify neck rotation, poor visualization, small distance from the spinolaminar junction to the posterior articular pillar line, or extreme lateral needle placement in an AP view [17]. The area spanning the spinolaminar line to the posterior articular pillar line is smaller in the lower cervical spine, and may be particularly diminutive at cervico-thoracic junction where epidural access is often undertaken.

In the thoracic spine, the spinolaminar junction is not well visualized, so the facet lucency is the principal landmark. The facet lucency is bounded by the superior lamina posteriorly and inferior lamina inferiorly. Given the slight angulation of the lamina, the spinolaminar junction (not well visualized) lies slightly posterior to the facet lucency; this is the earliest the epidural space may be encountered, and only when the needle is midline. Given the flat lamina in the thoracic spine, in practical terms, the epidural space is most often encountered in the facet lucency posterior to the dorsal margin of the inferior lamina. Nevertheless, one must start anticipating entry into epidural space slightly before the facet lucency is approached. The area just behind the facet lucency represents Zone 0, Zone 1 is the posterior part of the facet lucency, Zone 2 is the anterior half of the facet lucency, and Zone 3 lies anterior to that. Most of the time the point of entry into the epidural space lies in vicinity of Zones 1 & 2 [19].

In the lumbar spine the most proximate point at which needle tip may enter the epidural space is at the spinolaminar junction, in Zone 0 [18]. In the lumbar spine the distance from Zone 0 up to the facet joint lucency is Zone 1, the area spanning the facet lucency represents Zone 2, and the area beyond the facet joint lucency is Zone 3. All four zones are frequently encountered in the lumbar spine [18].

### 4.4 AP and lateral zones (transforaminal epidural reporting and other procedures using transforaminal approach, Fig. 5, Table 2)

Transforaminal epidural steroid injection (TFESI) is one of the most performed interventional spine procedures. The aim of the procedure is to deposit medications in the foramen at the site of pathology, which may involve spread to the ventral epidural space, the DRG, and proximal and distal exiting nerve root(s). Contrast spread patterns have been shown to correlate with needle tip location [5,6]. Multiple terminologies have been used to describe the technical aspects of TFESI needle positioning such as supraneural and infraneural, safe triangle, Kambin triangle, and preganglionic and postganglionic. For clinical and research purposes, it is important to describe the precise needle location as well as contrast spread pattern succinctly and clearly.

**Table 2:** Zones in AP and lateral view (transforaminal epidural access and other relevant procedures and reporting). For transforaminal epidural the terminology is based on the nerve residing in the foramen e.g., L4 TFESI represents needle placement at L4-5 neural foramen in proximity to the L4 nerve root. Reporting sequence (Mediolateral/Cephalo-caudal/antero-posterior dimension), whichever is missed an X is placed in its place.

AP View (lumbar & thoracic)	L (lateral 2/5 of the pedicle) green	6 (middle fifth of the pedicle) yellow	M (medial 2/5 of the pedicle) red
AP view (cervical)	L (lateral third of the pedicle) green	C (middle third of the pedicle) yellow	M (medial third of the pedicle) red
AP View cephalo-caudad^a^ (lumbar and thoracic)	S (upper 1/2) of the interpedicular line)	C needle position is indeterminate	I (lower 1/2 of the interpedicular line)
Lateral View^b^ (cephalo-caudad dimension)	S (upper 1/2) of the foramen	C needle position is indeterminate	I (lower 1/2 of the foramen)
Lateral View^b^ (antero-posterior dimension)	P (posterior 1/2) of the foramen	C needle position is indeterminate	A (anterior 1/2 of the foramen)

AP – anteroposterior, L – lateral, M −medial, C – central, S – superior, I - inferior.

a AP view used for cephalocaudal secondarily if lateral view not reported.

b Oblique in case of cervical with angulation from true AP noted.

In the AP view, the needle tip at the 6:00 O'clock position in relation to the pedicle is considered optimal for transforaminal epidural injections since it balances risk mitigation (e.g., intrathecal breach) and efficacy (i.e., obtaining epidural vs. solely nerve root spread). In addition to optimizing the risk: benefit ratio, the 6:00 O'clock position is the most prevalent landmark in the literature. Since there is considerable variation in the literature regarding the location of the DRG and extension of the dural sheath, as well as the position of the image intensifier relative to the spinous processes in patients with anatomical anomalies (e.g., a pure AP view might deviate significantly from 0° in patients with scoliosis), we concluded that the middle-fifth of the pedicle in an AP view corresponded to the center (designated ‘6’). Using the middle fifth as the central location allows the 6 O′ clock zone to represent a well-circumscribed midpoint, ranging from approximately 5.40–6.20 on the face of a clock. Anything lateral is referred to as ‘L’ and anything medial as ‘M’. Because of varying cephalo-caudad angles used for accessing the foramen, it is not necessary to describe needle tip position with the endplates aligned, as this is not standard practice and requires additional time and radiation exposure. In the lateral view, the needle position in the foramen is currently described using myriad terminologies; to simplify this we elected to divide the cephalo-caudad and AP dimensions into 2 equal parts. In the cephalo-caudad dimension, the upper half is designated as superior or “S” while the lower half is labeled as inferior or “I”. In the antero-posterior dimension in a true lateral view, the anterior half is designated “A” and the posterior half is “P”. We did not create a middle zone to minimize complexity. When the needle position is intermediate, best judgement should be employed to select one quadrant over the other, or the term central, “C” may be used. The AP position is reported first, followed by the cephalo-caudad location, followed by antero-posterior dimension in lateral view. Thus 6/S/A would denote the safe triangle. Whereas it is recommended that both AP and lateral view should be used to define the needle tip location, in cases where only the AP view is available, needle position can be described based upon its relationship to the pedicle in the AP view. In the AP view, the upper half of the distance between the inferior border of the upper pedicle and the superior margin of the lower pedicle is considered to be superior or ‘S’, the lower half is designated as inferior or ‘I’, and the term central or “C” may be used if the needle position is considered to be precisely at the midpoint. For example, using only an AP view, the needle could be described as 6/S/X (6 O'clock, superior) or L/I/X (lateral, inferior), with the first letter describing the AP position, the second describing the cephalo-caudal location and X denoting that the depth in lateral view is not reported. In the cervical spine there is no pedicle, so there is no 6 O'clock; instead, the articular pillar is divided into three equal zones: lateral (L), central (C) and medial (M). For the cervical transforaminal, the foramen is similarly categorized and divided as in lumbar spine lateral view, and the same methodology is followed except for the additional point of recording the obliquity of the fluoroscope from a true AP view. The AP zones are color-coded like traffic lights lateral to medial with green (go) representing Zone L, yellow (caution) representing Zone 6 or C, and red (stop and reassess) representing Zone M.

### 4.5 Reporting of medial branch blocks and radiofrequency neurotomy (Fig. 6, Table 3, Table 4)

The system used for reporting of medial branch blocks and radiofrequency neurotomy is for use in the lumbar and cervical regions. Because of the variability of location and innervation of thoracic medial branches, the nomenclature in this manuscript is limited to the low back and neck [[22], [23], [24]]. As evidence accumulates and further research clarifies precise targets for RFA, zones may be created accordingly.

**Table 3:** Lumbar medial branch block (MBB) and radiofrequency ablation (RFA) of medial branch. AP view. SAP – superior articular process.

Cephalo-caudad (AP)	Mediolateral (AP)	Lateral View
0-Base and side of SAP – ideal for RFA (green) 0 (for L5 dorsal ramus)- At the crevice between sacral articular process and ala (green)	L- lateral 2/5th of pedicle - ideal (green)	Posterior quarter of the neck of SAP (yellow) (excluding sacral SAP posterior quarter which is green)
1-side of SAP mid-upper SAP (yellow)	C- middle 1/5th of pedicle (yellow)	Middle half of the neck of SAP (green)
2- top of TP ideal for MBB (yellow)	M −medial 2/5th of pedicle (red)	Anterior quarter of neck of SAP (red)
3 - bottom of TP (red)

**Table 4:** Lateral cervical view for cervical medial branch block (MBB) and radiofrequency ablation (RFA) Lateral view. S – superior, C – central, I – inferior, A – anterior, M −middle, P – posterior.

Cephalo-caudad	Antero-posterior
S – upper third of articular pillar	A – anterior third of articular pillar (ideal for needle tip location for RFA)
C – central third of articular pillar (ideal for MBB & RFA except C7-T1)	M −middle third of articular pillar (ideal for MBB)
I – inferior third of articular pillar	P – posterior third of articular pillar

For the lumbar region, both a true AP view with the superior endplates overlapping at the targeted vertebral level and the lateral view showing the depth of insertion and relationship of the needle tip to the neck of the SAP are important. The relationship of the needle tip and shaft to the medial branch are especially important for radiofrequency lesioning. In the AP view, for location reporting, the most important landmarks are the superior articular process (SAP), and the superomedial aspect of the transverse process (TP) [25,26]. Needle tip Position 0 lies along the side of the SAP as it rises from the transverse process, covering the base and side of SAP, and is the ideal location for RFA; hence it is coded green. Position 1 lies higher than position 0 and maybe used for second lesions in the case of tall SAPs or insufficient electrical testing but creates a risk for inadequate ablation when used as a stand-alone ablation site, and hence is coded yellow. Position 2 centers over the superomedial aspect of the TP and this site may be used for a second lesion site in the case of a wide SAP or insufficient electrical testing, and hence is coded yellow. Position 2 is ideal location for medial branch blocks as it avoids spilling the local anesthetic over the ventral ramus and giving a false positive [25]. Position 3 creates the risk of inadequate lesion since the mammillo-accessory ligament may protect the nerve, and is thus coded red. In the mediolateral plane, Zone L covers the lateral 2/5th of the pedicle including the superomedial aspect of the transverse process and base of the SAP, and is coded green. Zone C covers the middle fifth of the pedicle and should serve as a cautionary line of restraint for advancement of the electrode too medially; hence, it is coded yellow. Zone M covers the medial aspect of the pedicle and is too medial for successful nerve ablation if placed dorsal to lamina, and in a position to cause neural trauma if placed ventrally; hence it is coded red.

For L5 dorsal ramus block and RFA, the needle tip position may be recorded at its target point in the crevice between the sacral articular process and ala, just lateral to the base of the superior articular pillar when the endplates are aligned. This area is approximately 2–4 mm in diameter and is designated as Zone 0. The needle tip may be described as in Zone 0, or inferior/superior or lateral/medial to it.

In the lateral view, the superior articular process serves as the major landmark for radiofrequency neurotomy. The angulation at each level varies according to anatomy. At all lumbar levels, the middle half of the width of the base of the SAP as it appears in the lateral view is coded green as it represents the ideal location. The anterior quarter is coded red and is to be avoided to not lesion the lateral and the intermediate branches. The posterior quarter is also suboptimal because the nerve may be protected by the mammillo-accessory ligament. For the L5 dorsal ramus, the anterior portion must still be avoided, but the posterior quarter is acceptable for lesioning as the mammillo-accessory ligament does not exist.

For cervical MBB and RFA, positioning the needle tip on the articular pillar is critical [27]. The lateral view with the articular pillars superimposed is ideal for reporting standards, and the needle tip location should be described first in the cephalo-caudad dimension and second in the AP dimension. The articular pillar may be divided into three zones in each plane with the endplates aligned, and the location of the needle tip is described in those planes as shown in Fig. 6. For third occipital neurotomy, lesions may be described as slightly above, on, and below the C2-3 joint line [28]. Because of technical considerations including the results of sensory and motor testing during ablative procedures (i.e., the reference standard for optimizing precision) and the variability of nerve locations, a uniform method of reporting nuances of this procedure may not be feasible.

For thoracic reporting, the variability of medial branches dictates that the procedure be reported individually [[22], [23], [24]].

### 4.6 Zones for disc and vertebral body procedures and anatomical reporting (Fig. 7, Table 5)

Discs and vertebral bodies are accessed in AP, oblique and lateral views; however, for final reporting, true AP and lateral views are most important. Common interventions for which this is useful include discography, disc injections, disc biopsies and regenerative medicine, annular ablations, percutaneous discectomy, vertebroplasty, kyphoplasty and basivertebral nerve ablation. In addition, this system may be useful for reporting pathology involving discs and vertebral bodies such as hemagiomas, tumors and fractures.

**Table 5:** Zones for disc and vertebral body reporting. R – right, M −middle, L – left, A – anterior, C- central, P – posterior, S – superior, C – central, I - inferior.

			
AP view^a^^d^	Right sided third(R)	Middle third (M)	Left Third (L)
Lateral View^b^	Posterior third (P)	Middle third (M)	Anterior Third (A)
Lateral View^c^ (Vertebral Body only)	Top third (S)	Middle third (C)	Lower third (I)

a AP view reported first.

b Lateral view reported second (depth).

c Cephalo-caudad reported last (vertebral body only).

d All disc zones except (MM) may further be divided and reported as annular “A” and nuclear “N”

The disc is initially divided into 3 equal segments in an AP view: left (L), middle (M), right (R). The horizontal plane is reported first. The disc is then divided into three segments in the lateral view, anterior (A), middle (M), posterior (P), which is reported second. Each segment except for MM can be further subdivided to account for the proportion containing annulus fibrosus and nucleus pulposus based on MRI or CT scans and denoted as "A” or “N”. The needle tip location may be defined by whether it is “A” or “N” within a segment and proportionality of nuclear to annular component of a segment may also be described except for “MM” which is 100% nucleus.

In addition to the system described for discs, the vertebral body can also be divided into three additional segments: superior (S), central(C), and inferior (I). Right, middle or left is reported first, anterior, middle, or posterior is reported second, and upper, central, or lower is reported last. Although this system creates more zones (n = 27), it is the simplest system that can account for 3 sections in 3 relatively large dimensions; an 8-zone system would create ambiguity for midline structures and be imprecise when precision reporting is necessary, such as during ablative procedures. When reporting injectate spread (e.g., vertebral augmentation) that may occupy many zones, the predominant ones can be separately specified. Whereas the system contains some redundancy e.g., in case of vertical body collapse, it nevertheless is intuitive and provides high precision with little ambiguity or complexity.

## 5 Conclusions

There is a clear need for standardized nomenclature when reporting procedural details for interventional spine interventions as well as for general spinal anatomical reporting. The first step is the development of such nomenclature as reported here. The second and more important step is ratification, leading to eventual adoption for clinical and research use. This will involve critical evaluation and dissemination by professional societies, clinicians, and teaching programs.

## Funding

There was no funding.

## Declaration of competing interest

The authors declare no conflict of interest.

## Figures and Tables

**Figure:**
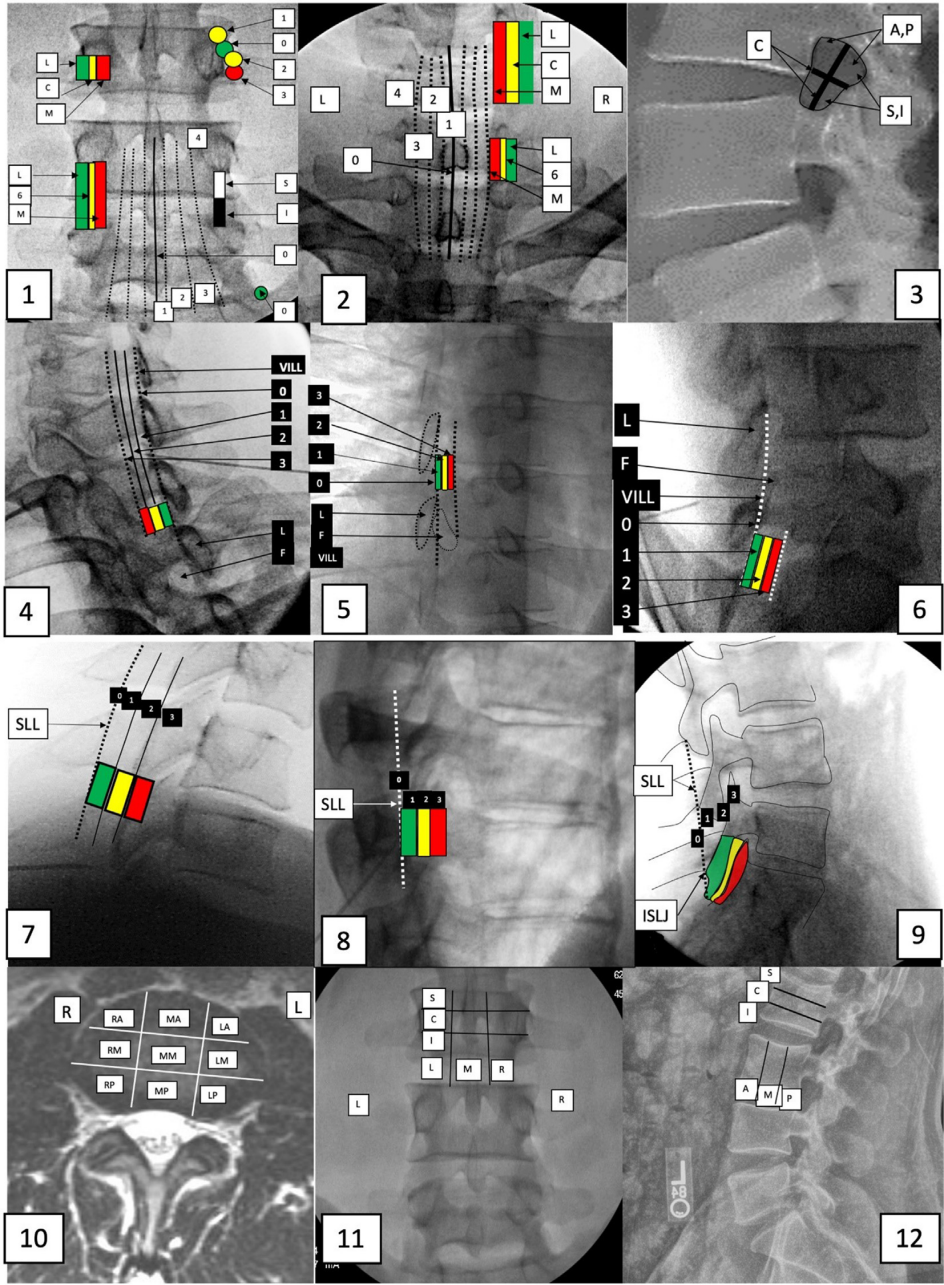


**Figure:**
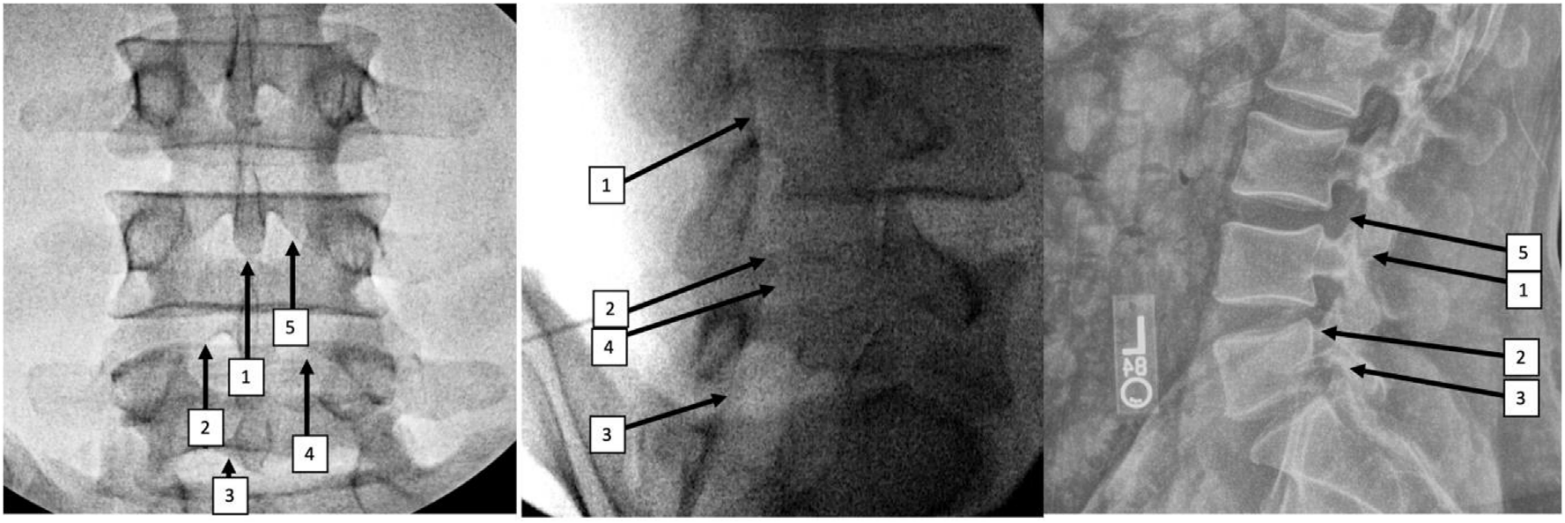


**Figure:**
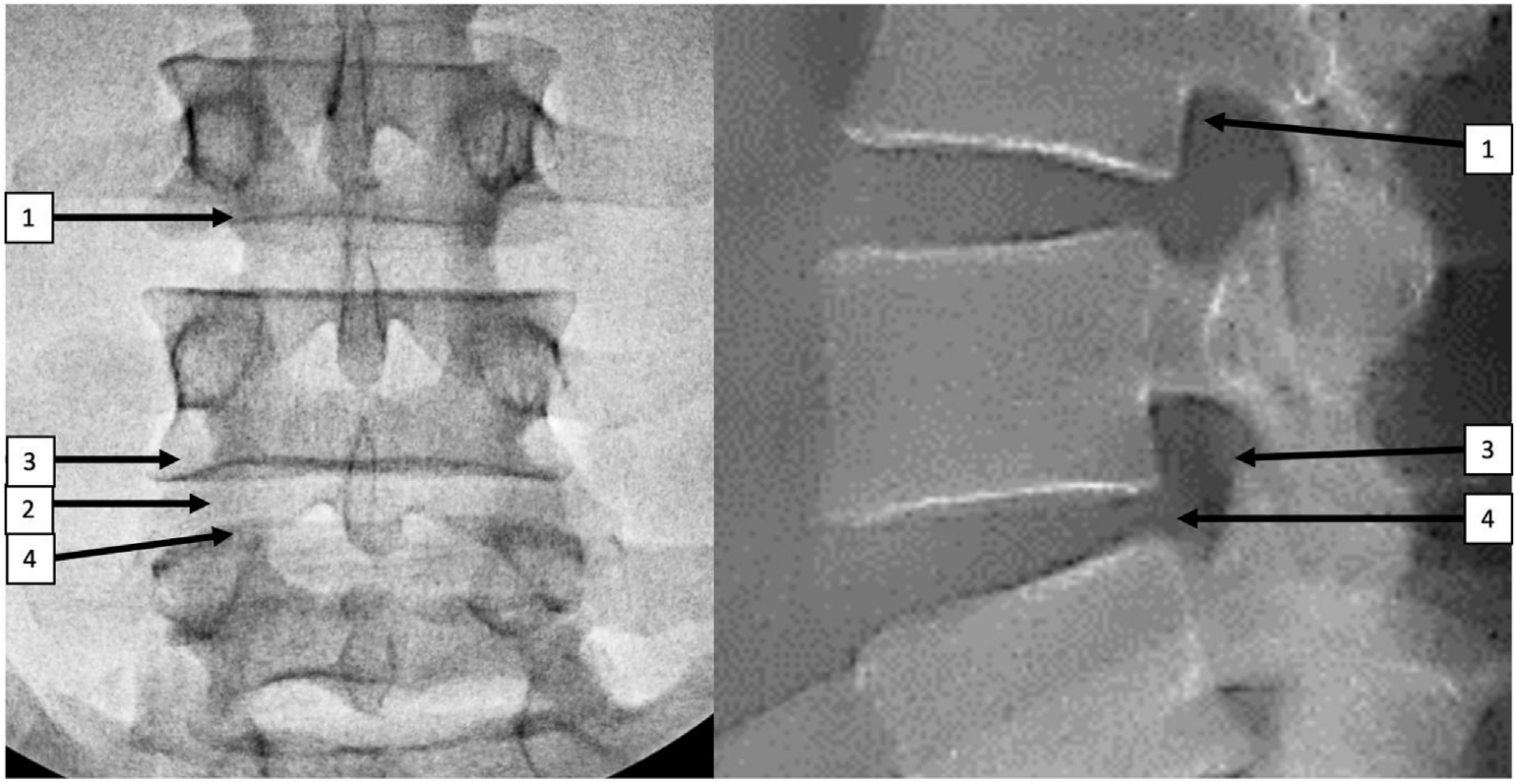


**Figure:**
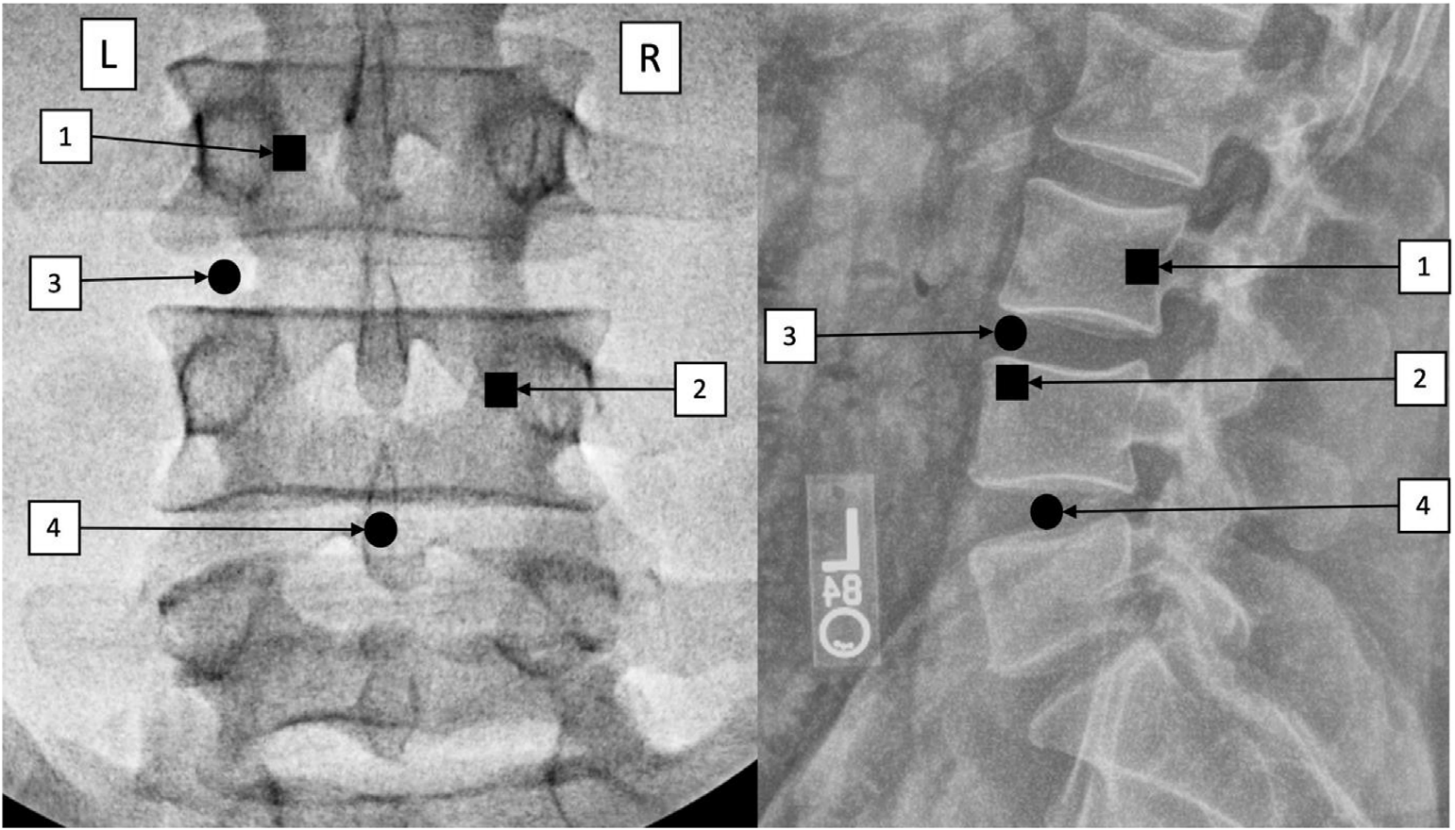


## Appendix A.

Summary Chart for Clinical Reference.

1. AP view lumbar spine with all the zones interlaminar and transforaminal and positions for lumbar MBB and MBRFA. *6 is the optimal position for transforaminal and 0 is optimal position for medial branch radiofrequency neurotomy (RFA).* Notice traffic light system with red zones “STOP” L - lateral, C – central, M – medial, S – superior, I – inferior.

2. AP view cervical and thoracic spine with all the zones interlaminar and transforaminal. Medial third (M) is red zone, “STOP”. M -medial, C - central, L – lateral.

3. Lateral view showing cephalo-caudad and antero-posterior dimensions. In case of ambiguity “C”, for central, can be used. *The cervical and thoracic foramen is divided in the same way. For the cervical foramen the view is not lateral but oblique and the degree of obliquity from the AP view is noted.* S - superior, I - inferior, A - anterior, P - posterior.

4. CLO view cervical. Zones 0-3. Green (go), yellow (caution), red (stop). Three equal zones lie between VILL and posterior articular pillar margin. *Zone 3 (red) should be rarely accessed at 50 degrees and posterior part of Zone 2 (yellow) may be seen occasionally with midline needles. Most epidural entry occurs in Zone 0 and 1.* VILL – ventral interlaminar line, L – lamina, F – foramen.

5. CLO view thoracic, Zones 0-3. Green (go), yellow (caution), red (stop). Three equal zones lie between VILL and anterior foramen margin. *Most epidural entry will occur in Zone 0-1.*

6. CLO view lumbar, Zones 0-3. Green (go), yellow (caution), red (stop). Three equal zones lie between VILL and anterior foramen margin. *Zone 2 seen with ligamentum flavum hypertrophy, Zone 3 is rarely accessed and may be seen with extreme lateral placement or severe ligamentum hypertrophy.*

7. Lateral view cervical Zones 0-3. Green (go), yellow (caution), red (stop). Zone 1 and 2 lie between spinolaminar junction and posterior articular pillar line. Zone 3 lies anterior to it. *Entry into epidural space will occur in Zone 1 and Zone 2. Zone 3 may be seen frequently when the space is obscure, head is rotated, and the distance from spinolaminar junction to articular pillar is diminutive or with extreme lateral needle placement.* SLL – spinolaminar line.

8. Lateral view thoracic Zones 0-3. Green (go), yellow (caution), red (stop). Zones lie from spinolaminar junction to beyond the facet lucency, Zone 3 *Entry into epidural space occurs at the facet lucency, Zone 1&2. SLL represents spinolaminar line marking most posterior location of the epidural space in true lateral view and lying slightly posterior to facet lucency.* SLL – spinolaminar line.

9. Lateral view lumbar Zones 0-3. Green (go), yellow (caution), red (stop). Zones lie from spinolaminar junction to beyond the facet lucency, Zone 3. *Entry into epidural space may be seen in all 4 zones but less frequent in Zone 0 and Zone 3. ISLJ represents inferior spinolaminar junction or the most posterior location where the epidural space may be encountered.* SLL – spinolaminar line.

10. T2 axial cut at the level of the disc showing divisions in thirds in AP and Lateral. *AP view is reported first, followed by lateral view.* R – right, L – left, A – anterior, P – posterior, M - middle.

11. AP view lumbar Spine showing disc and vertebral body zones. Divisions in thirds. S – superior, C – central, I - inferior.

12. Lateral view lumbar Spine showing disc and vertebral body zones. *AP view reported first, AP dimension in lateral view reported second, cephalo-caudad reported third and used only for the vertebral body*. A – anterior, M – middle, P – posterior, S – superior, C – central, I – inferior.

## Appendix B

### Key Points:

**Interlaminar Procedures and Reporting**

The AP view is reported first, followed by CLO view, and then followed by lateral view. Whichever view is not reported may be marked with an X.

**AP view**

1) Primarily four zones are described with midline being Zero. Same system is used in the cervical, thoracic and lumbar spine.

2) The end plates when aligned can be specified.

**CLO view**

1) Appropriate fluoroscope obliquity contralateral to the needle tip should be used. 50 degrees for cervical, 45 degrees for lumbar, and 60 degrees for thoracic

2) Lesser obliquity makes the needle appear deeper and vice versa

3) The entry into epidural space occurs at or slightly beyond (1–2 mm) the ventral interlaminar line.

4) Four zones (0-3) are reported at each spinal level with Zone 0 representing the ventral interlaminar line. A system like traffic lights is reported with green (Zone 1) meaning “go”, yellow, (Zone 2) meaning “caution”, and red, Zone 3, meaning stop and assess. With appropriate obliquity Zone 3 is accessed rarely, and at lumbar levels

**Lateral View**

1) Entry into epidural space may occur at the spinolaminar junction or beyond.

2) Four zones (0-3) are reported at each spinal level with Zone 0 representing the spinolaminar line or junction. A system like traffic lights is reported with green (Zone 1) meaning “go”, yellow, (Zone 2) meaning “caution”, and red, Zone 3, meaning stop and assess.

**Transforaminal Procedure reporting**

Needle tip is reported first in AP view, second in cranio-caudad (using lateral projection preferably), and third depth of insertion as seen in lateral projection. Whichever view is not reported may be marked with an X.

1) In the Lumbar and Thoracic spine in AP view the needle tip is reported in relation to the pedicle with middle 1/5th representing “6” 0 clock, and “L” meaning lateral 2/5th and “M” meaning medial 2/5th of the pedicle. Color coding with green (go) for “L”, yellow(caution) for “6” ideal position, and red for “M”- stop and reassess.

2) In the Cervical spine in the AP view, the articular pillars are divided into three equal zones. Lateral third is green (go), middle third is yellow (caution), and medial third is “red”, meaning stop and reassess.

3) The foramen is divided into superior and inferior quadrant, and anterior and posterior quadrant. Supero-inferior is reported second, depth is reported third. The supero-inferior may also be reported just by using the AP view. In case of ambiguity in either plane “C”, central, may be used. Same system is used for lateral view in lumbar and thoracic, and oblique view in cervical. The angle of obliquity (from AP) is preferably noted for the cervical transforaminal.

**Medial Branch Block and RFA reporting**

1) In the AP view the craniocaudad position of needle tip is described position 0-3 and reported first with 0 being the ideal position.

2) The mediolateral position is formed by dividing the pedicle in the same fashion as for transforaminal and reported as lateral 2/5^th^, “L”, middle 1/5^th^, “C” or medial 2/5^th^, “M”. Color coding system is followed with “M’ being red or stop.

3) The base of SAP is divided into middle half and two quarters anterior and posterior. Anterior quarter is red zone, middle half is green zone (ideal location), posterior half is yellow except at sacral SAP where it is green.

**Disc and Vertebral Body Reporting**

1) Disc is divided into three equal zones, right, “R”, middle, “M”, and left, “L” in AP view, and three in lateral anterior, “A”, middle, “M”, or posterior “P”. AP is reported first. Lateral is reported second. Location may further be clarified by “N” or “A” referring to nuclear or annular.

2) Vertebral body is further divided into three equal zones, superior, “S”, central, “C” or inferior “I” This dimension is reported last.

## Appendix C

### Examples:

#### A. Nomenclature for interlaminar access.

Interlaminar Examples.

The anatomical nomenclature is comprised of three numbers which represent the needle position identified in the AP, CLO and lateral fluoroscopic views.

1. Arrow (1), represents final position of a L3-4 interlaminar approach to the epidural space. In the AP view, the arrow (1) tip is in the center of the spinous process which is designated Zone 0. In the CLO view, the arrow (1) tip is at the VILL which is designated Zone 0.

In the lateral view, the arrow (1) tip is just before the facet joint lucency which is Zone 1. Thus, the 3-positional position of the arrow (1) tip is 0/0/1.

2. Arrow (2) represents final position of a L4-L5 interlaminar approach to the epidural space. In the AP view, the arrow (2) tip is left of the lateral edge of the spinous process and within the lateral half of the interlaminar opening which is designated Zone 3L where ‘L’ indicates the left side. In the CLO view, the arrow (2) tip is slightly past the VILL which is designated Zone 1. In the lateral view, the arrow (2) tip is beyond the facet joint lucency which is designated Zone 3. Thus, the 3-positional position of the arrow (2) tip is 3L/1/3.
3. Arrow (3) represents final position of a L5-S1 interlaminar approach to the epidural space. In the AP view, the arrow (3) tip is left of the lateral edge of the spinous process and within the medial half of the interlaminar opening which is designated Zone 2L. In the CLO view, the needle tip is at the VILL which is designated Zone 0. In the lateral view, the needle tip is in the area spanning the facet joint lucency which is designated Zone 2. Thus, the 3-positional position of the arrow (3) tip is 2L/0/2.
4. Arrow (4) represents final position of a L4-L5 interlaminar approach to the epidural space. In the AP view, the arrow tip is right of the lateral edge of the spinous process and within the lateral half of the interlaminar opening which is designated Zone 3R. In the CLO view, the arrow tip is past the VILL and within the middle portion of the area spanning posterior aspect of the VILL and the anterior margin of the observed foramina which is designated Zone 2. In the lateral view, the arrow tip is not visualized which is designated by the letter ‘X’. Thus, the 3-positional position of the arrow (4) tip is 3R/2/X.
5. Arrow (5) represents final position of a L3-L4 interlaminar approach to the epidural space. In the AP view, the arrow (5) tip is right of the lateral edge of the spinous process and within the lateral half of the interlaminar opening which is designated Zone 3R. In the CLO view, the orange arrow tip is not visualized which is designated by the letter ‘X’. In the lateral view, the arrow tip is beyond the facet joint lucency which is designated Zone 3. Thus, the 3-positional position of the arrow (5) tip is 3R/X/3.

#### B. Nomenclature for transforaminal access

Transforaminal Examples.

The anatomical nomenclature is comprised of a combination of three numbers and letters which represent the needle position identified in the AP view, the cephalo-caudad position in the lateral view (designated “S” for superior, “I” for inferior, and in case of ambiguity“C” for center), and the antero-posterior position in the lateral view (designated “A” for anterior and “P” for posterior). If the lateral view is not reported (designated by the letter “X”), then the needle position is described relative to the distance between the cephalad and caudad pedicles in the AP view where the upper half of the distance between the two pedicles is designated “S” for superior, the lower half is designated “I” for inferior and in case of ambiguity a center position is designated “C” for central.

1. Arrow (1) represents the final position of a left L3 transforaminal approach to the epidural space. In the AP view, the arrow (1) tip is at the 6 o'clock position relative to the pedicle which is designated 6. In the lateral view lateral cephalo-caudad view, the tip is in the cephalad position which is designated S. In the lateral antero-posterior lateral view, the tip is in the anterior position which is designated A. Thus, the position of the arrow (1) tip is 6/S/A.
2. Arrow (2) represents the final position of a left L4 transforaminal approach to the epidural space. In the AP view, the arrow (2) tip is at the 6 o'clock position of the pedicle which is designated 6. The lateral view is not reported. In the AP view, the tip is in the lower half of the distance between the cephalad and caudad pedicles which is designated I for inferior. Thus, the position of the arrow (2) tip is 6/I/X.
3. Arrow (3) represents the final position of a left L4 transforaminal approach to the epidural space. In the AP view, the tip is lateral to the 6 o'clock position of the pedicle which is designated L. In the lateral cephalo-caudad view, the tip is slightly superior which is designated S, but could also be termed C. In the lateral antero-posterior view, the tip is in the posterior half which is designated P. Thus, the position of the arrow (3) tip is L/S/P.
4. Arrow (4) represents the final position of a left L4 transforaminal approach to the epidural space. In the AP view, the tip is medial to the 6 o'clock position of the pedicle which is designated M. In the lateral cephalo-caudad view, the tip is in the inferior half which is designated I. In the lateral antero-posterior view, the tip is in the anterior half which is designated A. Thus, the position of the arrow (4) tip is M/I/A.

#### C. Nomenclature for vertebral body and disc reporting and procedures

Disc and vertebral body examples.

The nomenclature for vertebral procedures is comprised of three letters based on the AP and lateral view (AP and cephalo-caudad dimensions in lateral view). The nomenclature for disc is comprised of two letters based on the AP and lateral views, omitting cephalo-caudad.

1. Square (1) represents the final position for a vertebral body procedure or a location. In the AP view, the square (1) is left of midline which is designated L. In the lateral view, the antero-posterior position of the square (1) is posterior to the middle of the vertebral body which is designated P. Also in the lateral view, the cephalo-caudad position of the square (1) is in the center of the vertebral body which is designated C. Thus, the position of the square (1) is L/P/C.
2. Square (2) represents the final position for a vertebral body procedure or a location. In the AP view, the square (2) is right of midline which is designated R. In the lateral view, the antero-posterior position of the square (2) is anterior to the middle of the vertebral body which is designated A. Also in the lateral view, the cephalo-caudad position of the square (2) is in the superior portion of the vertebral body which is designated “S”. Thus, the position of the square (2) is R/A/S.
3. Dot (3) represents the final position for a disc procedure or location. In the AP view, the dot (3) is left of midline which is designated L. In the lateral view, the dot (3) is anterior to the middle of the disc which is designated A. Thus, the position of the dot (3) is L/A.
4. Dot (4) represents the final position for a disc procedure or a location. In the AP view, the dot (4) is in the middle of the disc which is designated M. In the lateral view, the dot (4) is in the middle of the disc which is designated M. Thus, the position of the dot (4) is M/M.
